# *Clonorchis sinensis* granulin: identification, immunolocalization, and function in promoting the metastasis of cholangiocarcinoma and hepatocellular carcinoma

**DOI:** 10.1186/s13071-017-2179-4

**Published:** 2017-05-25

**Authors:** Caiqin Wang, Huali Lei, Yanli Tian, Mei Shang, Yinjuan Wu, Ye Li, Lu Zhao, Mengchen Shi, Xin Tang, Tingjin Chen, Zhiyue Lv, Yan Huang, Xiaoping Tang, Xinbing Yu, Xuerong Li

**Affiliations:** 1 Department of Parasitology, Zhongshan School of Medicine, Sun Yat-sen University, Guangzhou, 510080 People’s Republic of China; 2 Key Laboratory for Tropical Diseases Control of Ministry of Education, Sun Yat-sen University, Guangzhou, 510080 People’s Republic of China; 3 Provincial Engineering Technology Research Center for Biological Vector Control, Guangzhou, 510080 People’s Republic of China; 4 Research Institute of Infectious Diseases, Guangzhou Eighth People’s Hospital, Guangzhou Medical University, Guangzhou, 510060 People’s Republic of China

**Keywords:** *Clonorchis sinensis*, Granulin, Cholangicarcinoma, Hepatocellular carcinoma, Immunolocalization, Cell migration

## Abstract

**Background:**

Long-term infections by *Clonorchis sinensis* are associated with cholangitis, cholecystitis, liver fibrosis, cirrhosis, and even liver cancer. Molecules from the worm play vital roles in disease progress. In the present study, we identified and explored molecular characterization of *C. sinensis* granulin (*Cs*GRN), a growth factor-like protein from *C. sinensis* excretory/secretory products (*Cs*ESPs).

**Methods:**

The encoding sequence and conserved domains of *Cs*GRN were identified and analysed by bioinformatics tools. Recombinant *Cs*GRN (r*Cs*GRN) protein was expressed in *Escherichia coli* BL21 (DE3). The localisation of *Cs*GRN in adult worms and *Balb*/*c* mice infected with *C. sinensis* was investigated by immunofluorescence and immunohistochemistry, respectively. Stable *Cs*GRN-overexpressed cell lines of hepatoma cells (PLC-GRN cells) and cholangiocarcinoma cells (RBE-GRN cells) were constructed by transfection of eukaryotic expression plasmid of pEGFP-C1-*Cs*GRN. The effects on cell migration and invasion of *Cs*GRN were assessed through the wound-healing assay and transwell assay. The levels of matrix metalloproteinase 2 and 9 (MMP2 and MMP9) in PLC-GRN or RBE-GRN cells were detected by real-time PCR (qRT-PCR). The levels of E-cadherin, vimentin, N-cadherin, zona occludens proteins (ZO-1), β-catenin, phosphorylated ERK (p-ERK) and phosphorylated AKT (p-AKT) were analysed by Western blotting.

**Results:**

*Cs*GRN, including the conserved GRN domains, was confirmed to be a member of the granulin family. *Cs*GRN was identified as an ingredient of *Cs*ESPs. *Cs*GRN was localised in the tegument and testes of the adult worm. Furthermore, it appeared in the cytoplasm of hepatocytes and biliary epithelium cells from infected *Balb*/*c* mouse. The enhancement of cell migration and invasion of PLC-GRN and RBE-GRN cells were observed. In addition, *Cs*GRN upregulated the levels of vimentin, N-cadherin, β-catenin, MMP2 and MMP9, while it downregulated the level of ZO-1 in PLC-GRN/RBE-GRN cells. In total proteins of liver tissue from r*Cs*GRN immunised *Balb*/*c* mice, vimentin level decreased, while E-cadherin level increased when compared with the control groups. Meanwhile, the levels of p-ERK reached a peak at 4 weeks post immunisation and the level of p-AKT did at 2 weeks after immunisation.

**Conclusions:**

The encoding sequence and molecular characteristics of *Cs*GRN were identified. As a member of granulin superfamily, *Cs*GRN induced mesenchymal characteristics of PLC and RBE cells and was found to regulate the activities of the downstream molecules of the ERK and PI3K/AKT signalling pathways, which could contribute to the enhancement of cell migration and invasion.

**Electronic supplementary material:**

The online version of this article (doi:10.1186/s13071-017-2179-4) contains supplementary material, which is available to authorized users.

## Background

*Clonorchis sinensis*, a human liver fluke, is a major food-borne parasite in China [1]. Long-term infections can lead to liver fibrosis, cirrhosis and even carcinogenesis [2, 3]. Some epidemiological studies and clinical researchers have shown that the incidence rate of primary hepatocellular carcinoma (HCC) is much higher in patients infected with *C. sinensis* than in non-infected patients [4–6].

A homologue of granulin from *C. sinensis* (*Cs*GRN) was identified in our previous studies, which was predicted to be a component of excretory/secretory products (ESPs) [7, 8]. Granulins are a family of secreted, glycosylated peptides that are cleaved from a single precursor protein with 7.5 repeats of a highly conserved 12-cysteine granulin/epithelin motif [9]. As independent growth factors, granulin family members are excessively expressed in various tumour tissues and are important in normal development, wound healing, and tumorigenesis [10]. Overexpression of progranulin (PGRN), which is a 60 to 90 kDa glycoprotein containing seven tandemly repeated granulin motifs in mammals, is linked to tumorigenesis in numerous human tissues, including liver. It is also associated with an aggressive and invasive tumour phenotype [11]. The *ov*-grn-1, a granulin among the ESPs of *Opisthorchis viverrini*, may establish a tumorigenic environment that ultimately manifests as cholangiocarcinoma (CCA) [12, 13]. FhGLM, a granulin-like molecule in *Fasciola hepatica* that is likely to be secreted through a nonclassical pathway, might exert a proliferative action on host cells during fascioliasis [14].

ESPs of *C. sinensis* (*Cs*ESPs) were reported as one of the most important factors for pathogenesis [15]. Our previously studies showed that some molecules from *Cs*ESPs could cause obvious apoptotic inhibition, and promote proliferation and migration of human HCC cells, which might exacerbate the process of HCC patients combined with *C. sinensis* infection [16, 17].

Given the close phylogenetic relationship with liver flukes and topologically similarity to both *ov*-grn-1 and PGRN, we proposed that *Cs*GRN may have a similar biological function of other granulin superfamily members as a growth factor. In the present study, the molecular characteristics of *Cs*GRN and its potential roles in the pathogenesis of clonorchiasis were investigated.

## Methods

### Parasite and preparation of *Cs*ESPs

Adult worms of *C. sinensis* were isolated from the bile ducts of infective cats. After washing procedures, the adult worms were used for tissue sections preparation or total RNA extraction. Adult worms were also cultivated to collect *Cs*ESPs and eggs, according to the method described previously [18].

### Bioinformatics analysis of the CsGRN sequence

The mRNA sequence annotated with granulin (*Cs*GRN) was selected from our *C. sinensis* cDNA plasmid library and was identified by DNA sequencing. The domains, physicochemical properties and some structures of the translated amino acid sequence were predicted with proteomics tools at NCBI (http://www.ncbi.nlm.nih.gov) and ExPaSy website (http://www.expasy.org/), and the disulphide bonds were analysed through the website (http://scratch.proteomics.ics.uci.edu/). The multiple alignments of sequences with homologues from human and helminths were carried out by Vector NTI software, and the phylogenetic tree was constructed with corresponding sequences from 14 other species using the software MEGA version 6.0 [19].

### Gene cloning, expression and purification of recombinant *Cs*GRN

The ORF of *Cs*GRN (GenBank KY855531) was 714 bp and the specific primers were as follows: forward 5′-CGC GGA TCC TGT AAA TAT AAC CAG ACT TG-3′ (*BamH* I) (Thermo Scientific, Waltham, USA), Reverse: 5′-TTA CTC GAG CGG AGC ACA GGT GTA GTG AT-3′ (*Xhol* I) (Thermo Scientific). The underlined bases indicated restrictions sites. cDNA was synthesised from total RNA, which was isolated from frozen *C. sinensis* adult tissues. The amplification conditions were 94 °C for 1 min, 55 °C for 1 min and 72 °C for 1 min for 30 cycles, plus 72 °C for 10 min. The purified PCR products were ligated into the pGEM-T-Easy vector (Promega, Madison, USA), followed by transformation into *E. coli* DH5α (Promega). The resulting plasmid DNA was digested with the appropriate restriction enzymes, ligated into the pET-28a (+) expression vector (Novagen, Darmstadt, Germany), and then transformed into *E. coli* BL21 (DE3) (Promega). Selected clones were grown and induced with 1 mM isopropyl-β-d-thiogalactoside (IPTG, Sigma, Guangzhou, China) at 20 °C for 12–18 h. The bacterial cells were collected by centrifugation and were sonicated on ice. The supernatant was collected, and recombinant protein was purified using the His-Bind Purification Kit (Novagen). The lysates of purified protein were subjected to 12% sodium dodecyl sulfate-polyacrylamide gel electrophoresis (SDS-PAGE). The concentration of purified recombinant protein was determined by using the BCA protein assay kit (Novagen).

### Preparation of antiserum for *Cs*ESPs and *Cs*GRN

The recombinant *Cs*GRN (r*Cs*GRN) and *Cs*ESPs were emulsified with complete Freund’ s adjuvant (Sigma-Aldrich, Guangzhou, China) and were subcutaneously immunised with 200 μg of protein for each rat initially. Subsequently, each rat was given 100 μg of protein (emulsified with equivalent incomplete Freund’s adjuvant (Sigma-Aldrich) for three booster injections at 2-week intervals. Anti-*Cs*GRN serum was collected every 2 weeks. Two weeks after the final boosting, the rats were sacrificed, and the sera were collected. Sera from naïve rats were also collected as a control. The antibody titers were determined by enzyme-linked immunosorbent assay (ELISA) and immunoblot analysis. For the production of mouse anti-*Cs*GRN serum, *Balb*/*c* mice were initially immunised with 100 μg of purified r*Cs*GRN followed by 50 μg the next three times, as described in the above method. The mice were sacrificed, and the liver tissues and serum were collected every 2 weeks from the final boosting for 8 weeks. The liver tissues and serum from normal mice during the same periods were also collected as a control.

### Immunofluorescence assay (IFA)

Freshly prepared *C. sinensis* adult worms were washed with phosphate buffer solution (PBS, 20 mM, pH 7.4) and then were fixed with 4% paraformaldehyde. The worms were dehydrated by a graded ethanol series, embedded in paraffin blocks and stored in a desiccator until use. Sections (4 μm thickness) were mounted on slide glasses, deparaffinized, rehydrated and rinsed with PBS. The slides were incubated with rat anti-*Cs*GRN or rat normal sera (1:50 dilutions) in PBS at room temperature for 2 h and then were washed with PBS three times. After incubation with Cy™3-conjugated anti-rat IgG (1:400 dilutions; Proteintech Group, Chicago, USA) for 2 h, the slides were washed with PBS three times and were observed under a light/fluorescence microscope (Olympus BX63, Hatagaya, Japan).

### Immunohistochemical localisation of *Cs*GRN

Female *Balb*/*c* mice (6–8 weeks of age) in the *C. sinensis* group were intragastrically infected with metacercariae (30 metacercariae per mouse). We determined the success of infection by stool examination. Five mice from each group were randomly selected and were sacrificed at 7, 30, 60, 90, 120 and 180 days postinfection (uninfected mice in same terms were used as controls). The liver tissues were extracted, and tissue sections were prepared. These samples were fixed and cut by a microtome of 4 μm sections. The sections were incubated with mouse anti-*Cs*GRN serum or mouse naïve serum (1:50 dilutions) overnight at 4 °C after being dewaxed in xylene, dehydrated in ethanol, and blocked with normal goat serum. The sections were washed with PBST (0.1% Tween-20 in PBS) and were incubated with horseradish peroxidase-conjugated goat anti-mouse IgG (1:400 dilutions; Proteintech Group) for 1 h. The sections were rinsed with PBS for 15 min, after which the slides were developed with diaminobenzidine (DAB). Next, the sections were counterstained with Mayer’s haematoxylin, dehydrated, cleared in xylene and mounted in PermountH. Images were captured with a microscope (Olympus BX51, Hatagaya, Japan).

### Cells culture, construction of the eukaryotic expression plasmid pEGFP-C1-*Cs*GRN and generation of stable cell line

RBE (human cholangiocarcinoma cell line, ATCC), and PLC (human hepatocarcinoma cell line, ATCC) were maintained as specified by ATCC protocols. RBE cells were cultured in RPMI 1640 (Gibco, Carlsbad, USA) while PLC was routinely maintained in DMEM medium (Gibco). These cell lines were supplemented with 10% fetal bovine serum (FBS, Gibco) and penicillin-streptomycin (100 units/ml) in 5% CO_2_ at 37 °C.

Standard molecular biology techniques were used for the construction of the pEGFP-C1-*Cs*GRN recombinant plasmid. The PCR product of the *Cs*GRN fragment was cut and inserted between the *Xhol* I (Thermo Scientific) and *EcoR* I (Thermo Scientific) restriction sites in the pEGFP-C1 vector (Promega). The forward and reverse primers (restriction sites are underlined) used to amplify this fragment included forward 5′-GCG CCT CGA GTG TAA ATA TAA CCA GAC-3′ (*Xhol* I) and reserve 5′-ATA AGG ATC CCG GAG CAC AGG TGT AG-3′ (*EcoR* I) respectively, based on the following conditions: 30 s denaturation at 94 °C, 30 s annealing at 60 °C, and 1 min extension at 72 °C for 30 cycles, plus 72 °C for 10 min. Cells (1 × 10^6^) plated in a 6-well cell culture cluster were transfected with either 0.8 μg of pGFP-C1 or pGFP-C1-*Cs*GRN using lipofectamine 2000 (Invitrogen, Carlsbad, USA) according to the manufacturer’s instructions. Two days after transfection, the stable cell line selection was started using the optimal concentration of G418. The medium was changed every 2–3 days, and the cells were split when necessary. After 2–4 weeks, all of the non-transfected cells disappeared, and isolated colonies began to appear. The selective overexpression of GFP or *Cs*GRN cells was designated as PLC-GFP/RBE-GFP cells and PLC-GRN/RBE-GRN cells, respectively. *Cs*GRN overexpression in these cells was checked by a fluorescence microscope, fluorescence activated cell sorting (FACS) analysis, qRT-PCR analysis and Western blotting first incubated with mouse anti-*Cs*GRN sera (1:100 dilution).

### Cell migration and invasion assay

To further confirm the role of *Cs*GRN in human cancer progression, wound-healing assays were performed to evaluate the effect of *Cs*GRN on cell migration as described previously [20]. The transfected PLC and RBE cells seeded in 6-well plates were grown to 80% confluence and were wounded by scratching with p200 pipette tips. Wounds were observed and photographed under a light microscope (Leica DMI3000B, Wetzlar, Germany) every 24 h for 72 h. The distances between the parallel cell edges were measured at each time point using Image J software. For each well, three different fields along the scratch were analysed in triplicate. Cell motility was measured as the percentage of the cell migration distance, which was regarded as the initial scratch distance. To evaluate the effect of *Cs*GRN on cell invasion, we performed transwell assays as described previously [21]. The PLC-GFP/RBE-GFP cells and PLC-GRN/RBE-GRN cells were suspended at 5.0 × 10^4^ per insert with serum-free media and then were transferred to wells filled with a culture medium containing 10% FBS as a chemoattractant. After 24 h of incubation, non-invading cells on the top of the membrane were removed with a cotton swab. The migrated cells on the underside of the filter membrane were fixed and stained with 0.1% crystal violet. The number of migrated cells on the membrane was counted in five randomly selected microscopic fields, and the cells were photographed. The protocol used for the invasion assay was the same as that used for the migration assay, except that the transwell insert was coated with Matrigel (BD Biosciences, Heidelberg, Germany).

### Real-time reverse transcription PCR

Total RNA of transfected cells was isolated using the Trizol reagent and was reverse transcribed to cDNA using ABM’s 5× All-In-One RT Master Mix (Transgen, Beijing, China). For qRT-PCR, SYBR Premix Ex Taq (Takara, Dalian, China) was used according to the manufacturer’s instructions. The primers were as follows: for *Cs*GRN, forward 5′-CGC GGA TCC TGT AAA TAT AAC CAG ACT TG-3′ and reverse 5′-TTA CTC GAG CGG AGC ACA GGT GTA GTG AT-3′; for MMP2, forward 5′-TAC AGG ATC ATT GGC TAC ACA CC-3′ and reverse 5′-GGT CAC ATC GCT CCA GAC T-3′;for MMP9, forward 5′-TGT ACC GCT ATG GTT ACA CTC G-3′ and reverse 5′-GGC AGG GAC AGT TGC TTC T-3′; for human actin, forward 5′-GGC ACT CTT AGC CTT CCT TCC T-3′ and reverse 5′-GCC AGA CAG CAC TGT GTT GGC GT-3′. Reactions were conducted under the following conditions: 95 °C for 30 s, 40 cycles of 95 °C for 5 s, and 60 °C for 20 s. The melting curves were analysed automatically by a collection of the fluorescence signals, and the expression of mRNA was calculated and normalised using the 2^-ΔΔCt^ method relative to actin with CFX96 software (Bio-Rad, Hercules, USA). Independent experiments were performed in triplicate.

### Western blotting analysis

*Cs*GRN (5 μg) and *Cs*ESPs (30 μg) were subjected to 12% SDS-PAGE and then were electro-transferred onto polyvinylidene difluoride (PVDF) membranes (Whatman, Maidstone, UK) at 100 V for 1 h in a Trans-Blot transfer (Bio-Rad). The membranes were blocked with 5% (w/v) skimmed milk in PBS for 2 h at room temperature and then were probed with mouse anti-His tag monoclonal antibody (1:2000, Novagen), rat anti-*Cs*GRN serum (1:200), rat anti-*Cs*ESPs serum (1:200) and rat naïve serum (1:200) at 4 °C overnight, respectively. After washing with PBS, the membranes were successively incubated with HRP-conjugated rabbit anti-rat or rabbit anti-mouse IgG at 1:2000 dilution (Proteintech Group) at room temperature for 1 h. The blots were visualised by enhanced chemiluminescence (ECL, Millipore, Billerica, USA). We also used Western blotting to detect the mechanism of cancer progression induced by *Cs*GRN. In detail, the total proteins of transfection cells were extracted using radioimmunoprecipitation assay lysis buffer (RIPA, Beyotime, Shanghai, China) and then were electrotransferred onto PVDF membranes. The membranes were incubated at 4 °C overnight with E-cadherin (1:2,000), vimentin (1:2,000), N-cadherin (1:2,000), ZO-1 (1:2,000), β-catenin (1:2,000), or GAPDH (1:2,000) diluted in blocking solution. In addition, the membranes with total proteins from *Balb*/*c* mice liver tissues were incubated at 4 °C overnight with E-cadherin (1:2,000), vimentin (1:2,000), N-cadherin (1:2,000), ZO-1 (1:2,000), β-catenin (1:2,000), p-ERK (1:2,000), ERK (1:2,000), p-AKT (1:2,000) or GAPDH (1:2,000) diluted in blocking solution, all antibody were products from cell signaling technology (CST, Boston, USA).

### Statistical analysis

Experimental data were obtained from three independent experiments with a similar pattern; data are expressed as the means ± standard deviation. The Student’s *t*-test and ANOVA were used to determine the statistical significance of the data obtained, and the means were compared between the groups using SPSS21.0 statistical software. A *P* < 0.05 represented a statistically significant difference.

## Results

### Sequence analysis

**Fig. 1:**
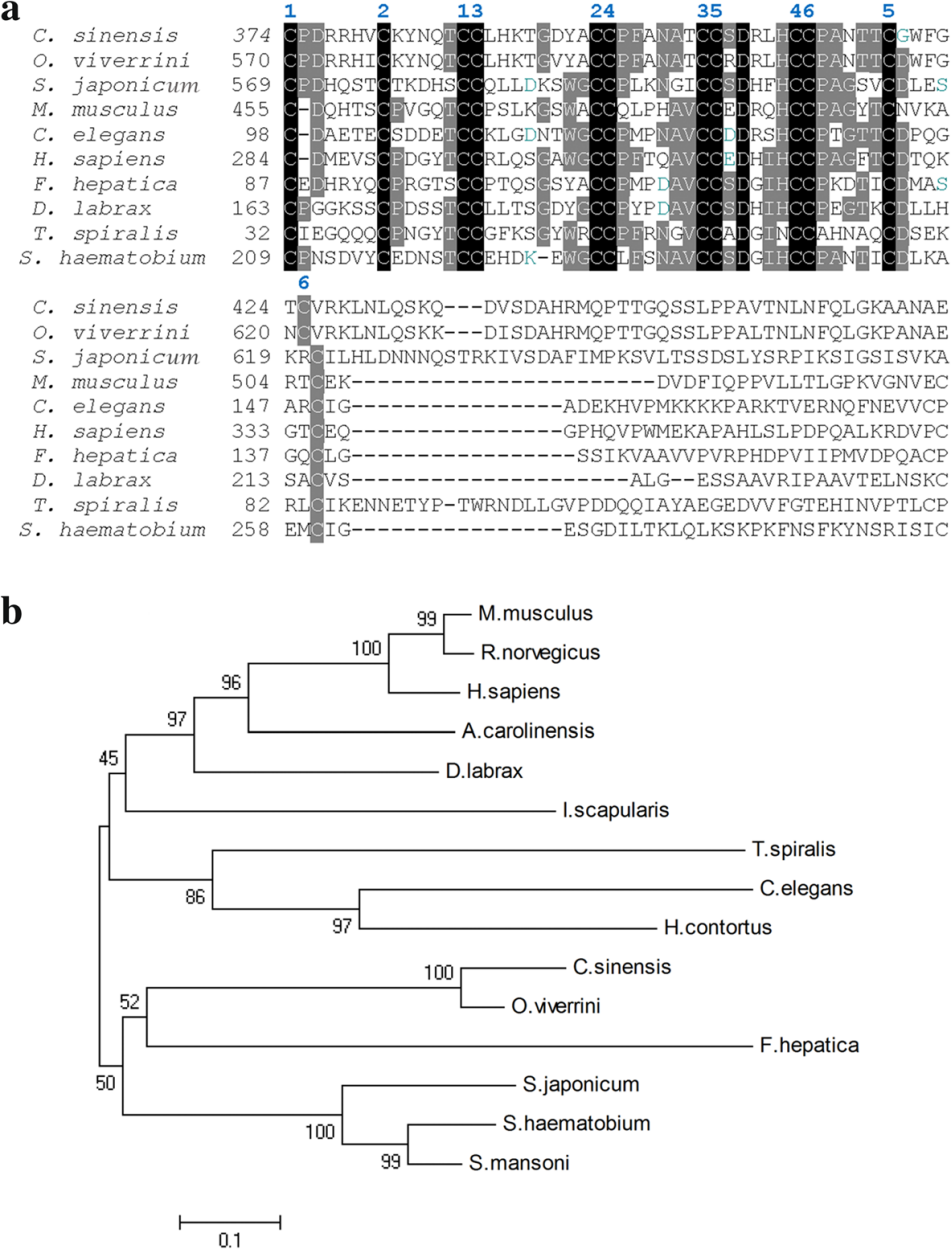
**a** Multiple alignment of sequences with one core granulin domain from various species are performed with Vector NTI software. Identical sequences are in *black* and conservative sequences are in *grey*; theoretical disulphide bonds are numbered one to six above each cysteine residue. **b** Phylogenetic tree for the granulins from a range of phyla constructed by MEGA 6.0 using the neighbor-joining method, Poisson correction is required and bootstrap values are 1,000. The abbreviations and accession numbers of the sequences are as follows: *Clonorchis sinensis* (*C. sinensis*, GAA54205.1); *Opisthorchis viverrini* (*O. viverrini*, XP_009174632.1); *Caenorhabditis elegans* (*C. elegans*, NP_492982.1); *Dicentrarchus labrax* (*D. labrax*, CBN81737.1); *Fasciola hepatica* (*F. hepatica*, ID BN1106_s891B000441 in the WormBase ParaSite); *Schistosoma japonicum* (*S. japonicum*, CAX73857.1); *Schistosoma haematobium* (*S. haematobium*, XP_012796138.1); *Homo sapiens* (*H. sapiens*, NP_002078.1); *Mus musculus* (*M. musculus*, NP_032201.2); *Rattus norvegicus* (*R. norvegicus*, AAH72469.1); *Ixodes scapularis* (*I. scapularis*, XP_002415868.1); *Trichinella spiralis* (*T. spiralis*, XP_003371171.1); *Anolis carolinensis* (*A. carolinensis*, XP_008111426.1); *Haemonchus contortus* (*H. contortus*, CDJ86608.1), *Schistosoma mansoni* (*S. mansoni*, CCD75903.1)

The sequence of *Cs*GRN encoding 238 amino acids was predicted with molecular weight 25.8 kDa, theoretical point isoelectric (PI) of 8.5. It contains two granulin domains from sequence released in the GenBank (GAA54205.1), which is presumed without N-terminal signal peptide. Multiple alignments of the sequences among GRN and nine members from other organisms (Fig. 1a) indicated that it belongs to the granulin family with a conservative domain with rich cysteine peptides. They share low sequence identity to each other except for *O. viverrini*. In addition, the sequence is abundant with cysteines being presumed to contain thirteen disulphide bonds. Moreover, the phylogenetic analysis (Fig. 1b) suggested that the granulin protein from *C. sinensis* has a very close relationship to *O. viverrini*. Furthermore, the evolution of the members from host and trematode or nematode may vary greatly.

### Cloning, expression and purification of r*Cs*GRN

**Fig. 2:**
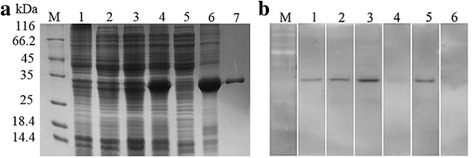
Expression and identification of r*Cs*GRN. **a** r*Cs*GRN was identified by 12% SDS-PAGE. Protein molecular weight markers (Lane M), lysate of *E. coli* containing pET-28a (+) without induction (Lane 1) and with induction by IPTG (Lane 2), lysate of *E. coli* containing pET-28a (+)-*Cs*GRN without induction (Lane 3) and with induction by IPTG (Lane 4), supernatant (Lane 5) and precipitate (Lane 6) of the lysate of *E. coli* containing the recombinant plasmid after induction, the purified recombinant *Cs*GRN protein (Lane 7). **b** r*Cs*GRN protein was identified as a component of *Cs*ESPs. r*Cs*GRN protein was probed by mouse anti-His-tag serum, rat anti-*Cs*GRN serum, rat anti-*Cs*ESPs serum and naïve serum (Lanes 1–4, respectively). In addition, *Cs*ESPs were probed with rat anti-*Cs*GRN serum and naïve serum (Lanes 5, 6). Protein molecular weight markers (Lane M)

The ORF of *Cs*GRN was cloned into the pET-28a (+) expression vector, and the recombinant plasmids were confirmed by restriction enzyme identification and DNA sequencing (not shown). The one mM IPTG-induced r*Cs*GRN was purified and analysed by SDS-PAGE, and the purified protein was obtained from the supernatant using the His-Bind Purification Kit (Fig. 2a).

### Identification of *Cs*GRN as a component of *Cs*ESPs

r*Cs*GRN was probed with mouse anti-His tag serum, rat anti-*Cs*GRN serum, rat anti-*Cs*ESPs serum, and rat naïve serum as a control (Fig. 2b, Lanes 1–4). In addition, *Cs*ESPs was probed with rat anti-*Cs*GRN serum (Lane 5) and rat naïve serum as a control (Lane 6). Rat anti-*Cs*GRN serum could specifically recognise *Cs*ESPs, and rat anti-*Cs*ESPs serum could react with r*Cs*GRN (Fig. 2b, Lanes 2, 5), confirming that *Cs*GRN was an excretory-secretory product of *C. sinensis*.

### Localisation of *Cs*GRN in parasites and infection *Balb*/*c* mouse liver

**Fig. 3:**
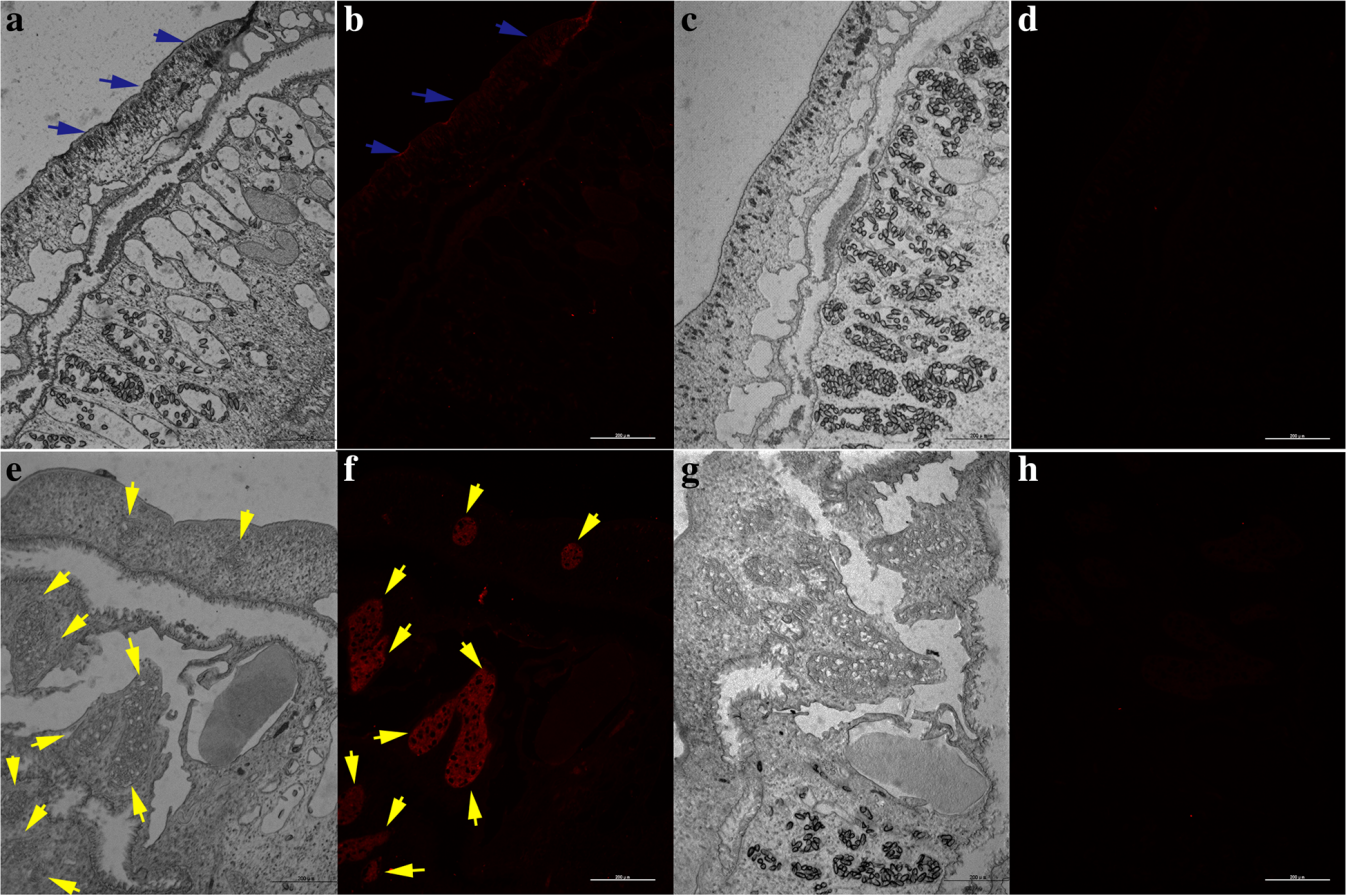
Immunolocalization of *Cs*GRN in *C. sinensis* adult worm. Sections of *C. sinensis* adult worm were probed with rat anti-*Cs*GRN serum (**a**, **b**, **e**, **f**) or normal rat sera (**c**, **d**, **g**, **h**) followed by my3-conjugated anti-rat IgG. Sections were imaged under fluorescence light (**b**, **d**, **f**, **h**), or white light (**a**, **c**, **e**, **g**). *Cs*GRN was distributed in the tegument and testes of the adult worm (**b**, **f**). *Blue arrow*: tegument; *Yellow arrow*: testes. The images were magnified at 100× (*scale*-*bar*: 200 μm) for adult worm

Immunofluorescence localisation analysis showed that *Cs*GRN was mainly localised in the tegument and testes of *C. sinensis* adult worm (Fig. 3).

**Fig. 4:**
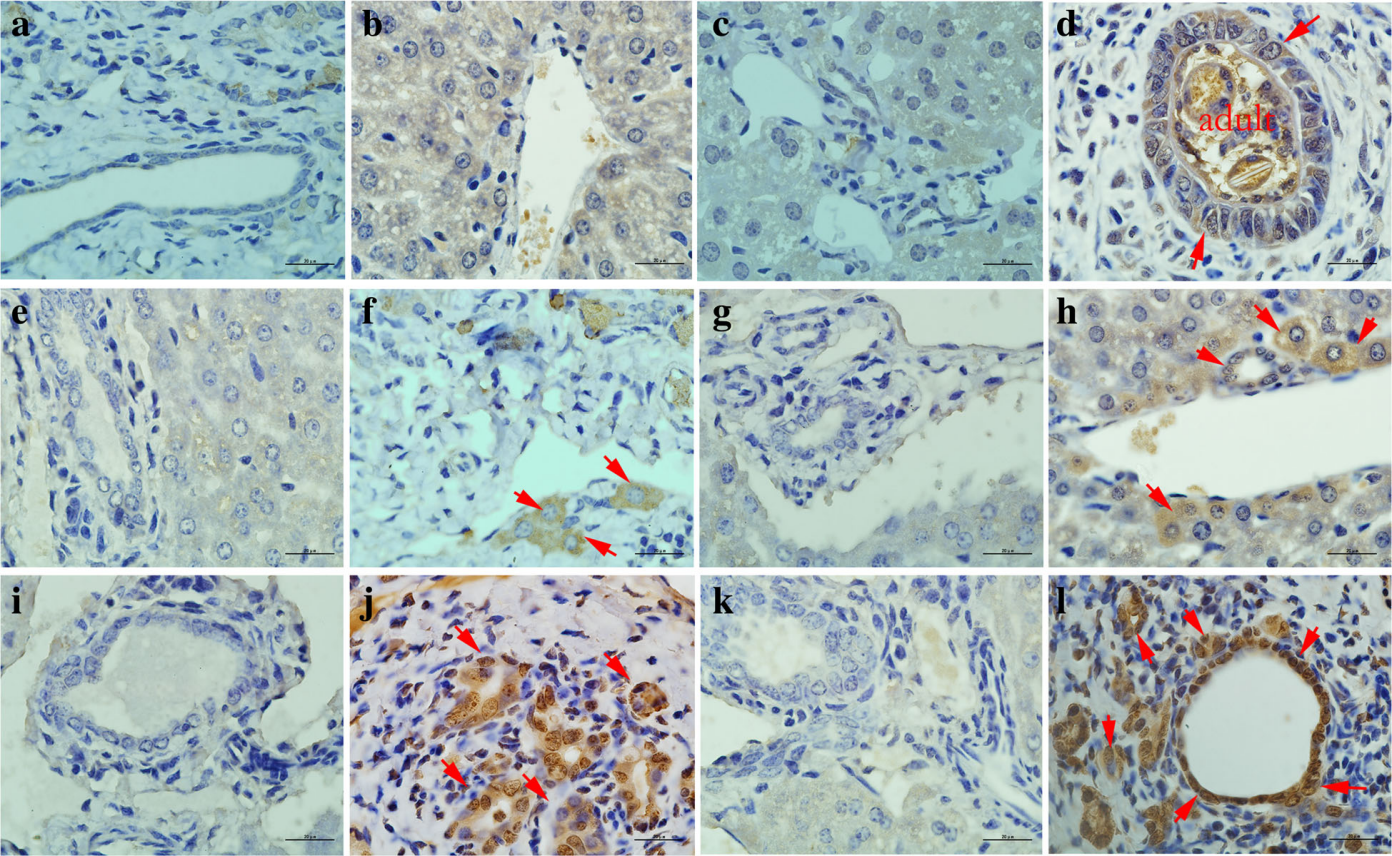
Immunohistochemical localisation of *Cs*GRN in infected *Balb*/*c* mouse livers. Sections of *Balb*/*c* mouse livers infected with *C. sinensis* treated with mouse anti-*Cs*GRN serum (**b**, **d**, **f**, **h**, **j** and **l**) or mouse naïve serum (**a**, **c**, **e**, **g**, **i** and **k**). During early infection (1 month), *Cs*GRN was observed mainly surrounding the bile ducts where parasites reside, and *brown* granules were found around cell nuclei, which explained how *Cs*GRN entered the biliary epithelium cells across the cell membrane (**d**). From 2 months post-infection, *Cs*GRN was also seen within the hepatocytes (**f**, **h**, **j** and **l**). With prolonged infection, more *Cs*GRN gathered in bile duct epithelial cells and hepatocytes. Positive hepatocytes and biliary epithelium cells were even found in the smaller bile duct where adult worm could not reach. **a**, **b** normal, **c**, **d** 1 month, **e**, **f** 2 months, **g**, **h** 3 months, **i**, **j** 4 months and (**k**, **l**) 6 months post-infection. Arrows indicate positive cells. The images were magnified at 1,000× (*scale*-*bar*: 20 μm)

The liver tissues from *Balb*/*c* mice infected with *C.sinensis* were analysed by immunohistochemistry using mouse anti-*Cs*GRN sera (Fig. 4). Liver sections from infected and uninfected mice probed with naive serum were not shown here. Positive staining was indicated with brown. Compared with sections from normal mice, strong staining was detected in the bile duct and the liver tissue after 1 month of parasite infection. Additionally, the level of *Cs*GRN was higher with extended time. Most importantly, brown granules were found around the cell nuclei, explaining that *Cs*GRN entered the biliary epithelium cells and hepatic cells across the cell membrane (Fig. 4). *Cs*GRN was also seen in the bile ducts that were distant from the liver flukes, while normal mouse biliary epithelium and liver tissues were unstained (Fig. 4a, c, e, g, i, k).

### *Cs*GRN overexpression in PLC-GRN and RBE-GRN cells

**Fig. 5:**
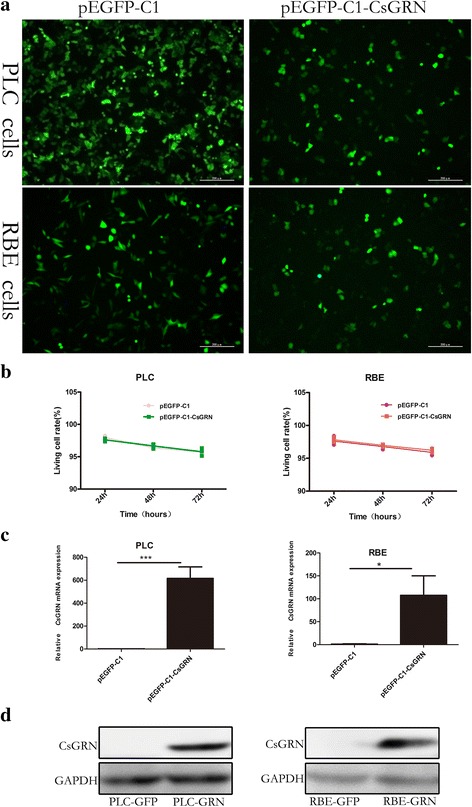
Successful construction of stable *Cs*GRN-overexpressed hepatoma cells and cholangiocarcinoma cells. **a** *Green* fluorescence was observed under an inverted microscope and was emitted by PLC and RBE cells transformed with pGFP-C1 and pGFP-C1-*Cs*GRN, respectively. The images were magnified at 100× (*scale*-*bar*: 200 μm).**b** Cellular viability of transfected PLC and RBE cells (24 h, 48 h and 72 h after transfection) was detected by Annexin V-APC/7AAD double staining with FACS analysis. **c** qRT-PCR was used to detect the expression of *Cs*GRN mRNA between the experimental group and control group. **d** Western blot analysis was used to determine the *Cs*GRN protein expression between the experimental group and control group. *P < 0.05, ***P < 0.001 compared with a matched group

The ORF of *Cs*GRN was cloned into the pEGFP-C1 eukaryotic expression vector, and the recombinant plasmids were confirmed by sequencing (Additional file 1: Figure S1). Viable cell images of most cells showed green fluorescence and grew well after the recombinant plasmid pEGFP-C1-*Cs*GRN transduction (Fig. 5a). To exclude the possibility that transfection plasmids could affect cell survival, the cellular viability of the transfected cells was detected by Annexin V-APC/7AAD staining with FACS analysis. Neither the pEGFP-C1 plasmids nor the pEGFP-C1-*Cs*GRN plasmids had any effect on cell viability (Fig. 5b). The qRT-PCR and Western blotting results showed the relative mRNA level and the protein expression of *Cs*GRN were significantly raised in the PLC-GRN and RBE-GRN cells compared with that in PLC-GFP/RBE-GFP cells (Fig. 5c, d), indicating that overexpressed-*Cs*GRN PLC/RBE cell line was successfully constructed.

### Cell migration and invasion triggered by *Cs*GRN

**Fig. 6:**
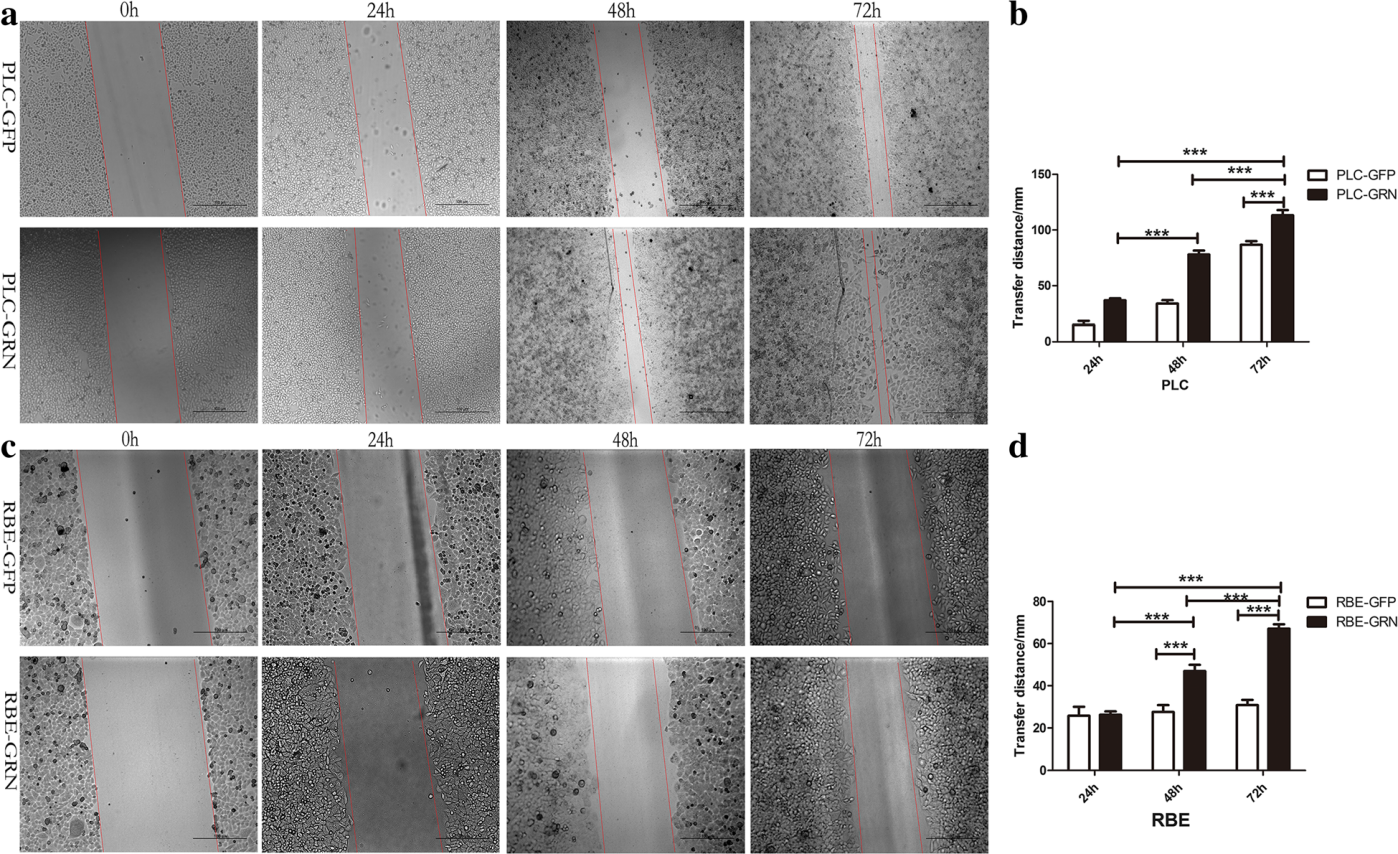
*Cs*GRN promotes increased cell migration. Cell migration of PLC and RBE cells shown by wound-healing assay (**a** and **c**). Cells were observed using light microscope under 5× objective (*scale*-*bar*: 100 μm). Assays were performed in triplicate. Relative cell migration level was calculated by normalising to cell migration level at 0 h. ***P < 0.001, compared with the control group. Data are presented graphically in panels **b** and **d**

**Fig. 7:**
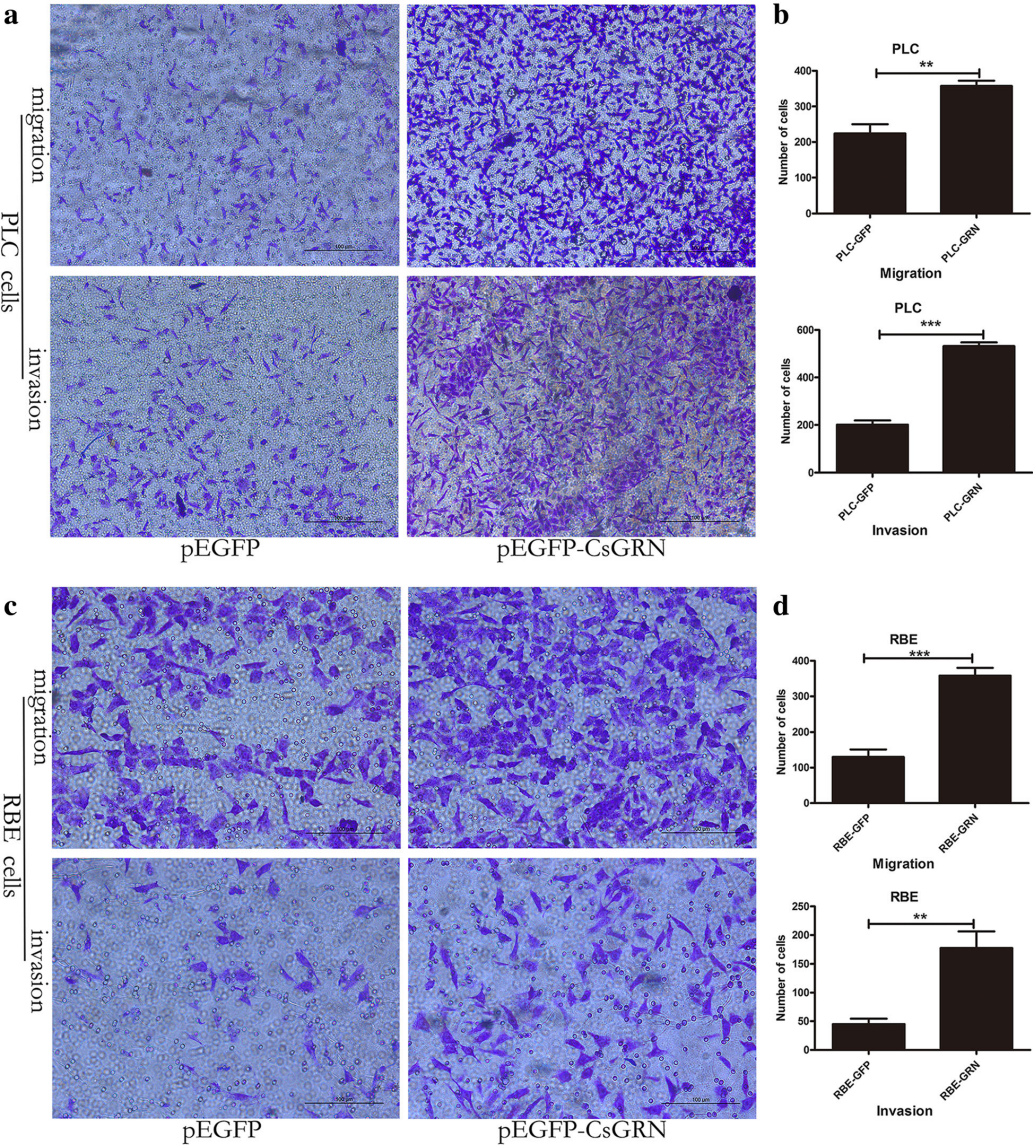
Cell migration and invasion triggered by *Cs*GRN in the transwell assay. PLC-GFP/GRN cells (**a**) or RBE-GFP/GRN cells (**c**) were suspended in serum-free media for 24 h. PLC-GFP cells and RBE-GFP cells were the negative control. Invasion assays were performed using Matrigel-coated membranes. The migration assay was similar to the invasion assay, except that the upper side of the membranes was not coated with the matrigel. Cells attached to the lower surface of the membranes at 24 h were counted under a light microscope. Cells were observed using a light microscope under a 10× objective (*scale*-*bar*: 100 μm). Ten random visual fields were selected to quantify the migration and invasion (**b** and **d**) using Image J software. **P < 0.01, ***P < 0.001, compared with control group

To determine whether *Cs*GRN could play any role in cancer cell migration, we carried out wound-healing assays. The results showed that PLC/RBE-GRN cells could induce significant cell migration when compared with PLC/RBE-GFP cells, respectively (Fig. 6). Similarly, transwell cell migration/invasion assay results indicated that the upregulation of *Cs*GRN in PLC and RBE cells significantly increased migration/invasive activity compared with that in the control group (Fig. 7). Therefore, *Cs*GRN could significantly improve the migration/invasion ability of CCA and HCC in vitro.

### Mechanisms of *Cs*GRN promoting CCA and HCC metastasis in vitro and in vivo

**Fig. 8:**
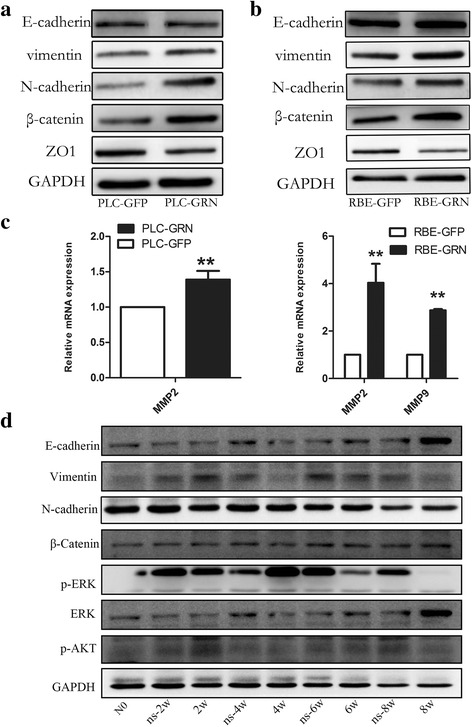
*Cs*GRN promotes mesenchymal characteristics. **a**, **b** Western blotting was used to detecting the expression of EMT-relevant markers in transfected PLC and RBE cells. PLC-GRN/RBE-GRN cells showed the upregulation of vimentin, N-cadherin and β-catenin, and downregulation of ZO-1; however, the expression level of E-cadherin had not changed. **c** Relative mRNA expression of MMPs in transfected PLC and RBE cells. MMP2 expression was higher in PLC-GRN cells, and MMP2 and MMP9 were higher in RBE-GRN cells than in PLC-GFP cells or RBE-GFP cells. **P < 0.01, compared with control group. **d** The expression of EMT-relevant markers and relevant signalling pathways indicators was detected in the liver from *Balb*/*c* mice injected with r*Cs*GRN protein. The level of E-cadherin increased, and the level of vimentin decreased in r*Cs*GRN protein-treated mice with an extended immune time extended (no change in N-cadherin). The expression levels of p-ERK and p-AKT were dynamically changed. p-ERK reached a peak in 4 weeks after immunisation, while p-AKT did at 2 weeks

To investigate the underlying mechanisms by which *Cs*GRN promotes CCA and HCC cell migration and invasion, Western blotting was performed to show the expression levels of epithelial-mesenchymal transition (EMT) relevant markers. The results indicated that overexpressed-*Cs*GRN PLC and RBE cell significantly increased vimentin, N-cadherin and β-catenin, and decreased ZO-1 compared with the control group, indicating that *Cs*GRN is involved in the EMT process in CCA and HCC cells (Fig. 8a, b). The relative mRNA expression of matrix metalloproteinases (MMPs) in transfected PLC and RBE cells showed that the MMP2 level was higher in PLC-GRN cells and that the MMP2 and MMP9 levels were higher in RBE-GRN cells than in PLC-GFP cells or RBE-GFP cells (Fig. 8c). To further validate the effects of *Cs*GRN on promoting cancer metastasis, we conducted animal experiments. By detecting the EMT-related indicators and relevant signalling pathway markers in *Balb*/*c* mouse liver immunised with r*Cs*GRN protein (Fig. 8d), we found that the level of E-cadherin increased, but vimentin decreased with the immune time extended. The signal indictors p-ERK and p-AKT were used to determine the mechanisms of migration. The expression levels of p-ERK and p-AKT were dynamically changed. In detail, p-ERK reached a peak in 4 weeks after immunisation while p-AKT did in 2 weeks. The statistical data of Fig. 8a, b, d are shown in Additional file 2: Figure S2.

## Discussion

Long-standing infections with *C. sinensis* eventually lead to clonorchiasis, which results in cholangiectasis, cholecystitis, cholelithiasis, hepatic fibrosis, and even CCA and HCC [22]. Additionally, researchers have proposed that mechanical damage caused by the activities of the parasites and *Cs*ESPs excreted and secreted from the liver fluke is the primary pathogenic mechanism [23]. In our previous studies [24], *Cs*GRN, one of an ingredient of *Cs*ESPs, was identified and the prokaryotic expression of the recombinant *Cs*GRN protein was carried out.

Sequence analysis showed that *Cs*GRN was a granulin member that has 28% overall identity with human PGRN. It shared the characteristic granulin motif that was reported to be a potent proliferative agent. Additionally, *Cs*GRN may promote carcinoma progression by prompting angiogenesis, insensitivity to apoptosis, tumour invasion and anchorage independence, all of which support tumour expansion in the unfavourable interstitial environment [25, 26]. Although the deduced protein was predicted without signal peptides, the localisation of *Cs*GRN in the tegument and testes of *C. sinensis* adult worm indicated that the protein could be secreted through other pathways such as that used by *F. hepatica* [14].

The protein was also found in the hepatocytes and cholangiocytes of infected *Balb*/*c* mice, possibly implying *Cs*GRN could be internalised by various mammalian cells via an endocytotic mechanism [27–29]. Moreover, the surprising finding provided a clue about the protein pertaining to its function on interaction with host cells as an ingredient of *Cs*ESPs. According to some reports, granulin plays a central role in the carcinogenesis of a range of malignancies [9]. For instance, PGRN is highly expressed in ovarian tumours [30], breast cancer [31], glioblastomas [32], and gastric cancer [33]. Moreover, as an important mediator of tumorigenesis and wound repair, it is associated with aggressive and invasive tumour phenotypes by stimulating invasion, protecting against anoikis, and supporting tumour expansion in the unfavourable interstitial environment [34, 35]. Based on these aspects, the overexpressed-*Cs*GRN stable cell lines named as PLC-GRN and RBE-GRN cells were successfully constructed, and the wound-healing assay and transwell assay were performed to observe the ability of the migration and invasion of CCA and HCC in vitro. The results displayed that they could stimulate cancer cell migration, which was similar to the action exerted by other recombinant proteins [36, 37]. Compared with cells co-cultured with protein, PLC-GRN and RBE-GRN cells were more approximate to the real situation that the internalised protein of this parasite might interfere with signalling and displayed the carcinogenic nature of biliary epithelial cells and hepatocytes. Likewise, *Helicobacter pylori* CagA protein targets PAR1/MARK kinase after delivery into gastric epithelial cells and disrupts cell polarity, resulting in disorganisation of the gastric epithelial architecture, inflammation and carcinogenesis [38]. Furthermore, similar to lipoprotein, internalisation induced by activating the fuel-sensing enzyme adenosine monophosphate-activated protein kinase (AMPK) is crucial to maintaining glioblastoma cell growth [39].

However, the mechanisms by which *Cs*GRN enhances tumour metastasis are unclear. And we hypothesised that they could be pertaining to EMT process, which endows cancer cells with a more aggressive phenotype, finally leading to tumour progression, including invasion and metastasis [40, 41]. Various biomarkers have been screened to demonstrate the EMT process, including the upregulation of mesenchymal markers such as N-cadherin, β-catenin and vimentin, as well as the loss of epithelial markers such as E-cadherin and ZO-1 [42].

Cadherins are transmembrane glycoproteins of cellular junctions that mediate calcium-dependent cell-cell adhesion and alterations in cadherin function have been implicated in tumorigenesis [43]. Although the loss of E-cadherin has been seen as a hallmark of EMT, recent evidence have indicated that a gain of expression of N-cadherin but without E-cadherin change in tumour cells contributes directly to an increased invasive potential and is independently associated with an early stage of metastasis [44, 45]. The catenins, serving to link the cadherin to the cytoskeleton, can regulate alterations in cadherin function [46]. Disruption of the connection between the cadherins and the cytoskeleton by mutations in β-catenin inactivates the adhesive function of E-cadherin in tumour cells and results in a non-adhesive phenotype. Thus, the increased expression of β-catenin is intimately related to the decline of E-cadherin [47]. Our experiments confirm these findings. Similarly, in PLC-GRN/RBE-GRN cells, the up-modulation of N-cadherin, β-catenin and vimentin, as well as the downregulation of ZO-1while the decrease of E-cadherin were not detected in vitro. Therefore, we can also draw the conclusion that *Cs*GRN induced the mesenchymal characteristics of PLC and RBE cells in responsible for cell invasion and metastasis. In contrast, in the liver of *Balb*/*c* mice immunised with r*Cs*GRN protein, the upregulation of E-cadherin and downregulation of vimentin indicated the block of EMT progress. In other words, anti-*Cs*GRN, a specific antibody produced by immunisation with r*Cs*GRN protein, probably prevents the tumour cells from invasion and metastasis.

As many studies have reported, MMPs play a predominant role in the process of tumour cell intravasation, and the dynamic interplay between N-cadherin and epidermal growth factor receptor (EGFR) leads to MMPs gene transcription [48]. As shown in our data, the expression of N-cadherin and MMPs was increased in PLC-GRN/RBE-GRN cells; therefore, we speculated that the level of MMPs was increased under the action of the N-cadherin and EGFR through stimulation by *Cs*GRN, eventually leading to the degradation of basement membranes and metastasis.

Numerous studies have shown that β-catenin, a key downstream effector in the Wnt signalling pathway, is considered to be a cell-cell adhesion protein, and most likely promotes tumour progression once activated [49]. We investigated the overexpression of β-catenin in vitro but without a change in vivo depending on our study, which prompted the speculation that β-catenin only acted as a biomarker of EMT but didn’t activate the β-catenin/wnt pathway.

To further study the potential downstream effectors modulated by *Cs*GRN promotion progression of HCC and CCA, we also tested the signalling molecules AKT and ERK using Western blotting. Our results showed that the expression of p-ERK and p-AKT were dynamically changed when p-ERK reached a peak in 4 weeks after immunisation while p-AKT did at 2 weeks. These data implied that the PI3K/AKT and ERK pathways participated in the promotion of EMT induced by *Cs*GRN, and they might not be activated at the same time.

As we know, the disease is often caused by complicated factors. This study observed that *Cs*GRN, as a *Cs*ESP like *Cs*CBs and *Cs*severin, has also been shown to promote cell migration and invasion. In consideration of the complex constitution of *Cs*ESPs, including proteases, antioxidant enzymes and metabolic enzymes [24, 50], interactions among these components are required to determine the authentic factors for pathogenesis in the future. We will explore the actions of granulin members with one or more typical domains in *C. sinensis* to clarify the difference between these molecules in our further studies.

Overall, we identified *Cs*GRN was a growth factor of *C. sinensis* and a vital constituent of *Cs*ESPs. To date, we have observed the potential promotion cell migration and invasion of *Cs*GRN. We also showed that the EMT process can be triggered by *Cs*GRN and involved in HCC and CCA metastasis via the activation of the PI3K/AKT and ERK pathways.

## Conclusions

In summary, we identified *Cs*GRN as belonging to the granulin family through bioinformatics and phylogenetic analyses. We also expressed and purified soluble r*Cs*GRN in *E.coli* and discovered it was an important component of *Cs*ESPs. In adult worm, *Cs*GRN is mainly located in the tegument and testes, which might be involved in parasite growth and development or even in the pathopoiesis of the parasite. *Cs*GRN could be detected at a high expression level in clonorchiasis-induced *Balb*/*c* mouse liver tissues and was even observed in hepatocytes and cholangiocytes. In addition, the secretory eukaryotic expression vector pEGFP-C1-*Cs*GRN was generated and employed to intervene in the expression and secretion of *Cs*GRN in PLC and RBE cells. An enhancement of cell migration and invasion was observed, and the current results suggested that *Cs*GRN is likely to promote cell migration and invasion by inducing liver EMT via the ERK and PI3K/AKT signalling pathways. The present study supports the involvement of *Cs*GRN in the pathogenesis of CCA and HCC.

## Additional files

Additional file 1: Figure S1. Successful construction of the eukaryotic expression plasmid pEGFP-C1-*Cs*GRN. **a** Restriction enzyme identification of the recombinant plasmid pEGFP-C1-*Cs*GRN. DNA ladder 5000 (Lane M), double enzyme digestion of pEGFP-C1-*Cs*GRN (Lane 1), recombinant plasmid pEGFP-C1-*Cs*GRN (Lane 2), empty vector pEGFP-C1 (Lane 3). **b **Sequencing data from recombinant plasmid pEGFP-C1-*Cs*GRN and *Cs*GRN gene were completely matched. (TIF 206 kb)

Additional file 2: Figure S2. Densitometric analysis of genes from Fig. 8**a**, **b**, **d**. Densitometric results were analysed with Image J software. Statistical comparisons between more than two experimental groups were made with one-way ANOVA tests followed by Tukey’s multiple comparisons test. Results are reported as the mean ± standard error of the mean (SEM), and *P* was set to 0.05. For all analyses, Prism 5.0 software (Graph Pad Software, San Diego, USA) was used. a *P < 0.05, **P < 0.01, compared with the control group. b *P < 0.05, **P < 0.01 and ***P < 0.001, indicate difference from experimental treatment. ^##^ *P* < 0.01 and ^###^ *P* < 0.001, compared with the matched pair. (TIF 910 kb)

## Abbreviations

AMPK: Adenosine monophosphate-activated protein kinase
CCA: Cholangiocarcinoma
*Cs*GRN: *Clonorchis sinensis* granulin
EGFR: Epidermal growth factor receptor
EMT: Epithelial to mesenchymal transition
ERK: Extracellular signal-regulated kinase
ESPs: Excretory/secretory products
HCC: Hepatocellular carcinoma
MMP2 and MMP9: Matrix metalloproteinase 2 and 9
MMPs: Matrix metalloproteinases
PGRN: Progranulin
PI3K: Phosphatidyl inositol 3-kinase
