# Supplementary figures and images for: Clonorchis sinensis granulin: identification, immunolocalization, and function in promoting the metastasis of cholangiocarcinoma and hepatocellular carcinoma
Source: Parasit Vectors. 2017 May 25;10:262. doi: 10.1186/s13071-017-2179-4 (PMC5445496; doi:10.1186/s13071-017-2179-4)

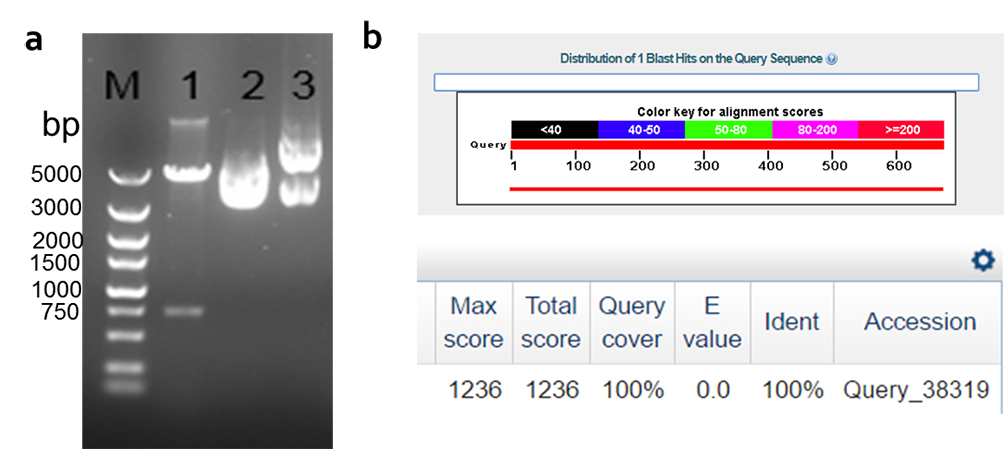

Supplement: Supplementary file 1 — Successful construction of the eukaryotic expression plasmid pEGFP-C1-CsGRN. a Restriction enzyme identification of the recombinant plasmid pEGFP-C1-CsGRN. DNA ladder 5000 (Lane M), double enzyme digestion of pEGFP-C1-CsGRN (Lane 1), recombinant plasmid pEGFP-C1-CsGRN (Lane 2), empty vector pEGFP-C1 (Lane 3). b Sequencing data from recombinant plasmid pEGFP-C1-CsGRN and CsGRN gene were completely matched. (TIF 206 kb) [file 13071_2017_2179_MOESM1_ESM.tif]

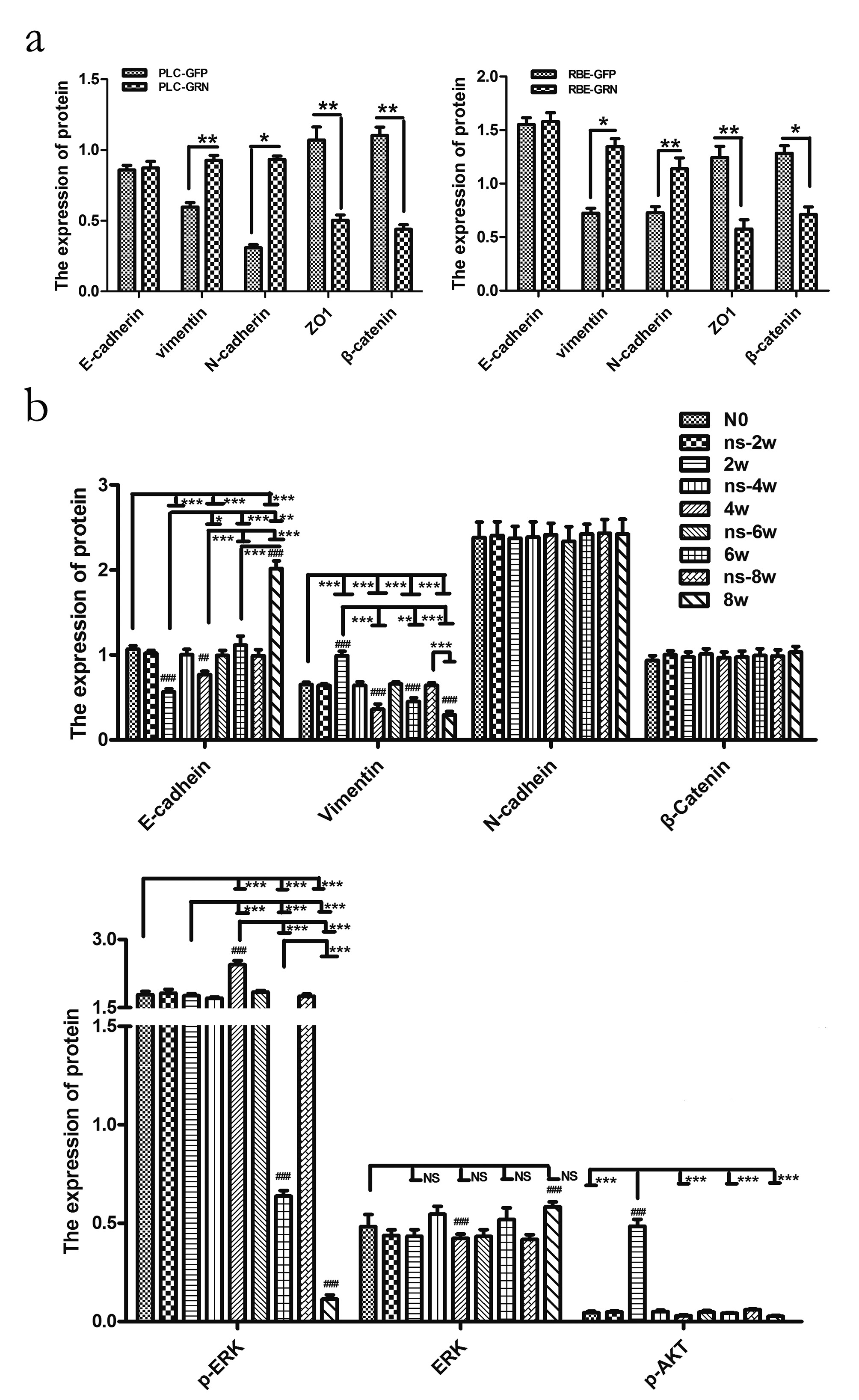

Supplement: Supplementary file 2 — Densitometric analysis of genes from Fig. 8a, b, d. Densitometric results were analysed with Image J software. Statistical comparisons between more than two experimental groups were made with one-way ANOVA tests followed by Tukey’s multiple comparisons test. Results are reported as the mean ± standard error of the mean (SEM), and P was set to 0.05. For all analyses, Prism 5.0 software (Graph Pad Software, San Diego, USA) was used. a *P < 0.05, **P < 0.01, compared with the control group. b *P < 0.05, **P < 0.01 and ***P < 0.001, indicate difference from experimental treatment. ## P < 0.01 and ### P < 0.001, compared with the matched pair. (TIF 910 kb) [file 13071_2017_2179_MOESM2_ESM.tif]
